# Drug Repurposing for Prevention and Treatment of COVID-19: A Clinical Landscape

**DOI:** 10.15190/d.2020.18

**Published:** 2020-12-16

**Authors:** Md. Shahadat Hossain, Ithmam Hami, Md. Sad Salabi Sawrav, Md. Fazley Rabbi, Otun Saha, Newaz Mohammed Bahadur, Md. Mizanur Rahaman

**Affiliations:** Department of Biotechnology and Genetic Engineering, Noakhali Science and Technology University, Noakhali-3814, Bangladesh; Department of Microbiology, University of Dhaka, Dhaka-1000, Bangladesh; Department of Applied Chemistry and Chemical Engineering, Noakhali Science and Technology University, Noakhali-3814, Bangladesh

**Keywords:** COVID-19, SARS-CoV-2, phases, clinical trial, vaccine, therapeutics, drug repositioning

## Abstract

SARS-CoV-2, the novel coronavirus strain responsible for the current pandemic of COVID-19, has rendered the entire humanity suffering. Several months have passed since the pandemic has struck. However, the world is still looking for an effective treatment plan to battle the viral infection. The first vaccine just received emergency approval in December 2020 for use in USA and UK. These are excellent news, however, the worldwide distribution of such vaccine, the possibility of virus mutation and the lack of data regarding the long-term effects of such vaccines are a significant concern. In addition, although remdesivir was recently approved by the FDA to be used as a clinical drug against COVID-19, it hasn’t stood out yet as a proven form of therapeutics. Such inability to produce a novel therapy has caused enough inconveniences for the affected people worldwide. Repurposing the already available drugs to fight against the virus seems to be a reasonable option amidst such uncertainty. Given the vast collection of potential treatment candidates to be explored against COVID-19, there is a decent chance that a success in this regard will serve the intermediary purpose of clinically treating the infection until a COVID-19 vaccine is widely distributed worldwide and will be able to treat COVID-19 patients that do not adequately respond to vaccines. Such treatments may prove very useful in future coronavirus outbreaks too. Proper research into these repurposing treatments may yield a certain insight into the field of novel treatment production as well. This review study accumulates a relevant set of information about drugs and vaccines against COVID-19, in terms of their repurposing properties and the specific phases of clinical trials they are undergoing across the world.  A potential timeline is also suggested to estimate when an effective result can be expected from the ongoing clinical trials for a better anticipation of the drug landscape. This study will hopefully help accelerate investment of resources into development and discovery of drugs and vaccines against the infection.

## Summary

1*. Introduction*

*2. History of COVID-19 and its Treatment*

*3. Methodology and Sources*

*4. COVID-19 Drug R&D Landscape*

*5. Types of Treatments in Clinical Trial*

- *5.1 Biological Treatments*

- *5.2 Chemically Derived Drugs*

*6. Vaccines*

*7. Countries and Sponsors Involved in Drug Trials*

*8. Timeline and Clinical Trial Phases*

*9. Conclusion*

## 1. Introduction

SARS-CoV-2, a new coronavirus strain detected in Wuhan City, Hubei Province, China is the infectious agent behind the coronavirus disease, 2019 (COVID-19), for which no efficient treatment is available to date^1,2^. This inability to produce a cure is surprisingly consistent with no approved medication being present for previous two major coronavirus outbreaks (SARS-CoV in 2003 and MERS-CoV in 2015) from the same genus^3,4^. An effective vaccine is important in order to obtain herd immunity^5^. The first vaccine just received emergency approval in December 2020 for use in USA and UK. However, it will take months to have it distributed worldwide, its long-term effects are hard to predict, and the possibility of future resistant mutations is a concern. In addition, because of novelty of the SARS-CoV-2 strain and its constant mutative nature, it is hard to synthesize a universal vaccine, so soon enough to be used across the globe right now^6,7^. In fact, SARS-CoV-2 has affected almost all the countries and provinces in the world and needs immediate attention by extensive approaches to sidestep the symptoms and the disease in a steady manner^8,9^. Having that in focus, repurposed drugs may serve as an intermediate purpose of treating the disease until a stronger novel drug or an universal vaccine is developed. Repurposed drugs simply work following a manipulated mechanism in already approved drugs for other conditions directed towards weakening the SARS-CoV-2 in a system^10^. Research into such an area requires a landscape of probable drug candidates to be observed on a regular basis. Now, a good number of drug companies, industries and other institutions around the world are investing into developing aptly repurposed drugs to tackle this global crisis. Here, we have compiled information about 876 clinical trials of various drugs that are currently being researched through tests to be finalized as drug candidates. We have categorized the drugs in terms of their nature to serve a better understanding of the purposes of each drug and the duration the drugs may require completing the clinical trial. By a comparative arrangement, we have presented a dynamic view of drugs to figure out their true potentials.

## 2. History of COVID-19 and its Treatment

SARS‑CoV‑2 belongs to the subfamily Orthocoronavirinae, family Coronaviridae, order Nidovirales. It comprises of four subtypes with two among them, named α and β, infecting humans, whereas the other two, named γ and δ, more likely to infect animals. It is only the seventh coronavirus known to infect humans until now and only the third to have caused a severe outbreak after SARS-CoV and MERS-CoV. Other coronaviruses, such as HKUI, NL63, OC43 and 229E manifest with milder symptoms compared to the ones in Betacoronavirus genus^11,12^. Being zoonotic in nature, it is believed that coronaviruses spread to humans from animals. Two cases were previously reported where human infections resulted in drastic disease. The first one was the 2002-2004 SARS outbreak, when humans got infected via the betacoronavirus named SARS-CoV, which is usually found in bats, resulting in 8,422 infections and 916 deaths around the world^13^. The second one was also produced by a betacoronavirus named MERS-CoV, initiated in Saudi Arabia in 2012, affecting about 3,000 people with concomitant 858 deaths^14^. The outbreak was well controlled at that time, although the virus came back a couple of times, causing separate outbreaks in South Korea (2015) and in Saudi Arabia (2018)^15^. MERS and SARS outbreaks never turned into pandemics because of two principal reasons. The first reason is that the reproductive value or R of both SARS-CoV and MERS-CoV were too low (2 or 3 for SARS CoV and below 1 for MERS-CoV), which means that from one infected person two or three more could be infected further. Hence, they were less contagious and avoiding close contact alone could suppress the possibility of being exposed to the diseases^16,17^. Unfortunately, in the case of SARS CoV-2, the R is around 4, which makes it more intimidating than any other viruses, despite having weaker manifestations^18^. The second reason is that the affinity of SARS-CoV-2 for angiotensin-converting enzyme 2 (ACE2) receptor is higher than that of SARS-CoV.

As far as drug R&D is concerned, several desperate attempts have been made to treat the COVID-19 since the onset of the emergence of the virus in China. Apparently, the very first approach was made through traditional Chinese medicine therapies (TCM) back in China in December 2019. Although none of those medications were designed exclusively for COVID-19, YinHuQingWen decoction and ShuFengJieDu capsules were suggested in preliminary case treatment with no effective result^19^. Implementation of systemic corticosteroids following the example of SARS‑CoV to treat pneumonia associated with COVID-19 showed, at least initially, no benefits either^12^. World Health Organization (WHO) constituted an R&D blueprint to draw suitable therapeutic methods to respond to the virus that included design and development of drugs, vaccines and other clinical techniques. Accompanied by European Medicines Agency, US Food and Drug Administration (FDA) and the Chinese government itself, WHO has already initiated materializing their goals by assembling resources and researchers to delve into establishing efficient vaccines and drugs.

## 3. Methodology and Sources

We retrieved information from the register of *www.clinicaltrial.gov* in 4 different categories: “Not yet recruiting”, “Recruiting”, “Enrolling by invitation” and “Active, not recruiting”. We considered going through drugs in all the study phases available including the non-applicable ones with no designation. Since we were studying clinical trials for the landscape, we emphasized on interventional study type of drugs. In addition to this systematic algorithm, we sought assistance from regular and contemporary literature reviews on drugs and vaccines landscape, in order to stay updated with our approach.

## 4. COVID-19 Drug R&D Landscape

**Figure 1:**
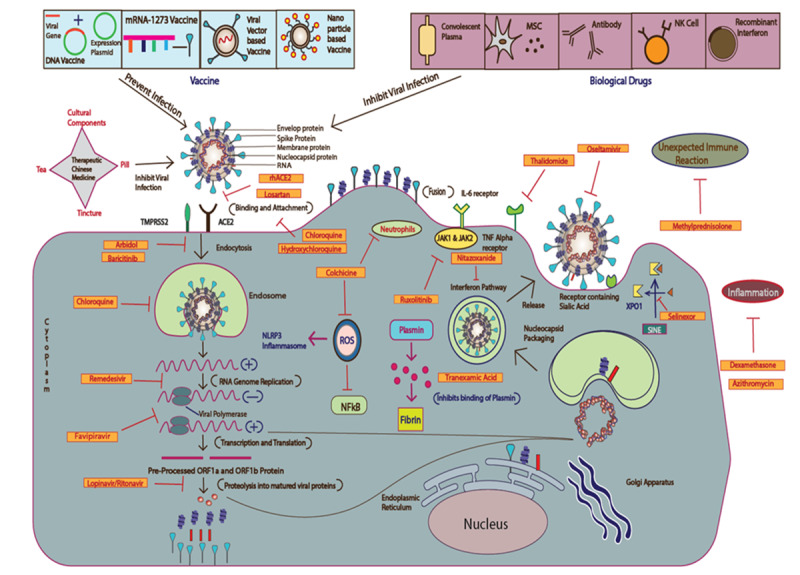
Overview of host pathways and viral replication mechanisms of the repurposed therapeutic drugs undergoing clinical trial against COVID-19 Drugs of both biological and chemical origins are shown along with a number of vaccines involved in repurposed applications in context of their respective pathways and mechanisms.

To date, resources are being invested more in the reinforcement of repurposing drugs suiting to COVID-19 cases than in developing novel drugs. That is because of: firstly, synthesizing a novel drug to combat the pandemic at the present time is highly unlikely to serve any purpose considering the unavailability of one perfect design or strategy and also requirement of a huge amount of time. Rather, to make the best use of existing drugs by manipulating them to fight against COVID-19 seems a much smarter approach, if it works. Secondly, in some cases of repurposing drugs, some clinical phases may not be required (phases I and II). Therefore, the drugs may become available on the market faster as compared to the novel ones. Thirdly, both pharmaceutical supply chains and distribution are readily available for such drugs. Besides, applying such drugs in combination with another drug may prove to be much more effective than monotherapy. And the last but not the least, drug repurposing may lead to discovery of new mechanisms of action for old drugs and may pave a way for a new target-based therapeutic in the process as well (**Figure 1**).

## 5. Types of Treatments in Clinical Trial

**Figure 2:**
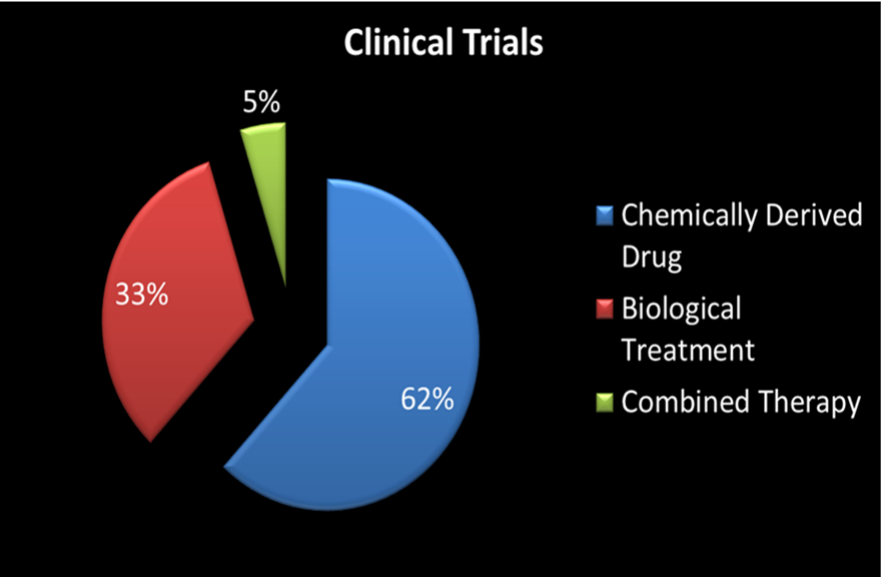
Percentage of chemically derived drugs, biological treatments and combined therapy in current clinical trials for repurposing against COVID-19 The graph has been generated using the Microsoft Excel application.

In current clinical drug research, as of 5^th^ of July, 2020, 62% of trials involve chemical, 33% biological and the remaining 5% include the combination of both chemical and biological drugs in nature (**Table 1, **Figure 2****).

**Table 1:** Total number of clinical trials for drugs of chemical, biological and combinatorial nature obtained from www.clinicaltrial.gov on 4^th^ of July, 2020

Treatment Types	Clinical Trials
Chemically Derived Drugs	542
Biological Treatments	294
Combined Therapy	42

### 5.1 Biological Treatments

**Figure 3:**
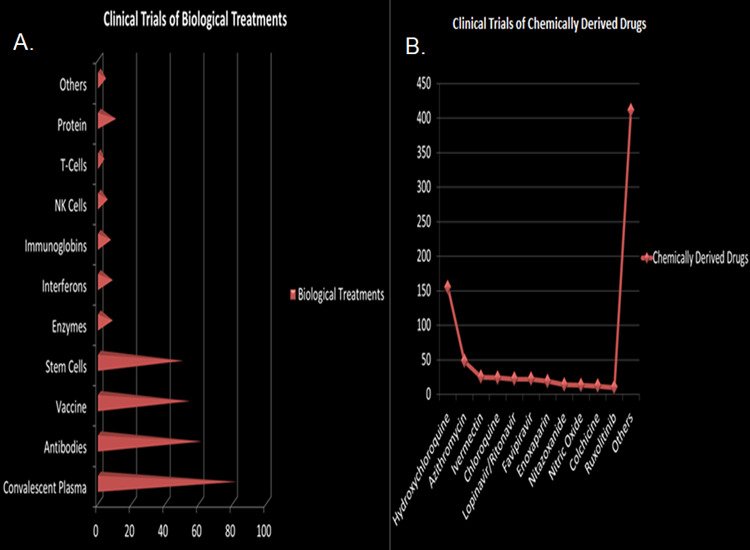
List of both (A.) biological treatments including vaccines and (B.) chemically derived drugs with their respective clinical trial numbers Vaccines mentioned with clinical trial numbers include both SARS-CoV-2 vaccines and different other vaccines which are being tested for repurposing purposes. Drugs of chemical nature with most occurrences in clinical trials are listed in orderly fashion. The graphs have been generated using the Microsoft Excel application.

Among the biological treatments (**Figure 3**A), antibody-mediated ones are mostly prevailing, especially tocilizumab and sarilumab being widely used in clinical trials. Convalescent plasma-based treatments are also being evaluated in clinical trials in large facilities, where plasma from cured patients is transferred to the infected patients. Another potential biological target for drug development is the use of stem cell. Several types of mesenchymal stem cells are now being tested as a treatment for COVID-19 patients. Other prospective biological drugs in clinical trials include interferons and NK cells. Even enzymes and peptide-based drugs are under consideration for decisive clinical trials (**Table 2**).

**Table 2:** Clinical trials using biological treatments

Biological Treatments	Clinical Trials
Convalescent Plasma	82
Antibodies	61
Vaccines	54
Stem Cells	50
Enzymes	8
Interferon	8
Immunoglobin	7
NK Cells	5
T-Cells	3
Protein	9
Others	4

#### 5.1.1 Convalescent Plasma

Convalescent plasma treatment involves transfusion of plasma content enriched with antibodies from a system exposed to a particular pathogen^20^. For decades now, this technique has served as a successful treatment for a rather short term recovery in individuals^21^. It works prophylactically for infected patients in preventing severity of the disease^22,23^. The mode of mechanism of the treatment is to take out the pathogen by cytotoxicity, phagocytosis or neutralization through propelling the antibodies to bind with the pathogens^24^. Convalescent plasma had already been used against SARS and MERS-CoV before^25^. South Korea and Taiwan conducted successful studies on convalescent plasma positively impacting severe cases of SARS and MERS-CoV^26,27^. As for SARS CoV-2, China facilitated a limited study on COVID-19 patients that exhibited a clinical improvement in terms of fever, cough and other symptoms^28,29^. A recently published article by Shen et al. (2020) reported improved respiratory status, viral loads and pulmonary lesions in patients administered with convalescent plasma leading to a meaningful recovery. However, no conclusive remarks on the efficacy of the therapy were made by the author. The result of another single ongoing clinical trial to determine the efficacy of anti-SARS-CoV-2 inactivated convalescent plasma remains to be seen^30^. Many clinical trials are currently being registered to observe effects of convalescent plasma in COVID-19 recovery (NCT04432013, NCT04388410, NCT04397757 etc).

#### 5.1.2 Recombinant Interferon

Virus infected cells naturally secrete type-1 interferon^31^. Combined with other drugs or even alone, interferon can demonstrate a diverse antiviral quality against multiple viruses, such as HCV, respiratory syncytial virus^32^, and α2β has already been declared to have inhibitory effects on both MERS-CoV and SARS-CoV^33^. Thus, numerous clinical trials are focusing on the safety and feasibility of the interferon treatment program for COVID-19 management (NCT04293887). Few other clinical trials on peginterferon products are under consideration in United States (NCT04344600, NCT04388709, NCT04331899, and NCT04343976) and in Canada (NCT04354259).

#### 5.1.3 Mesenchymal Stem Cell (MSC)

Stem cell research has been on the table for quite a long time now and mesenchymal stem cell (MSC) is being evaluated extensively for COVID-19. MSCs are basically anti-inflammatory agents that produce paracrine factors to repair damaged tissues and reduce pro-inflammatory cytokines at the same time^31^. Preclinical trials demonstrated that MSCs coupled with the ability of decreasing inflammatory infiltrate serve to retain endothelial permeability^34^. Immunomodulating effects of mesenchymal stem cells have already been proven to be effective on avian influenza viruses^35^. Presently, MSCs extracted from dental pulp and umbilical cord are being experimented for clinical use against COVID-19 (NCT04293692, NCT04269525, NCT04288102, NCT04302519)^31^. A study by University of Utah on MSCs out of amniotic fluid is in the earliest stage now (NCT04319731).

#### 5.1.4 Antibodies & Immunoglobulin

Pathogen specific antibodies as a part of humoral immune response have always been considered a formidable solution against viral infections. Screening cells and manufacturing specific antibodies for this purpose are rather time-consuming and require incessant labor, yet have been massively successful against the West Nile Virus^36^. Thus, it is obvious that producing SARS CoV-2 surface specific and epitope-targeting antibodies should be the sustainable and long term solution for COVID-19^37^. AbCellera from Canada and Eli Lilly and Company from USA are collaborating to develop a functional antibody for neutralizing SARS-CoV-2 from around 500 potential antibody sequences, by screening over 5 million immune cells from one of the early COVID-19 patients in USA^31^. A significant number of clinical trials focused on antibodies is being conducted, with more emphasis than on other biological drugs (NCT04344782, NCT04348500, NCT04409509, NCT04359901). Intravenous immunoglobulin (IVIG) also exploits a dynamic range of effects on the immune system depending upon doses applied^31^. As for the low doses of 0.2-0.4g/kg, IVIG works as a replacement therapy in antibody deficiency cases, whereas for higher doses of 2g/kg, IVIG displays its immunomodulating features by inhibiting inflammatory cells proliferation, antibody-dependent cytotoxicity and suppressing phagocytosis^38^. However, contemporary clinical trials against COVID-19 are focusing only on the low doses of IVIG (NCT04261426)^31^. Several clinical trials were performed to determine the effectiveness of human immunoglobulin in patients with pneumonia caused by 2019-nCoV^33^,including one by the Versailles Hospital (NCT04403269) in France and in Spain (NCT04432324).

#### 5.1.5 Natural Killer Cells (NK Cells)

Until today, the highest mortality caused by COVID-19 is seen among elder patients, which to some extent, may be related to the decreasing immune system with age. Therefore, efforts should be made to speed up the innate anti-viral immune response^39^. Natural killer (NK) cells are important parts of innate immunity system to elicit immediate response against viral infections^31^. The NK group 2 member A (NKG2A) receptors basically reduce mechanism of action of NK cells to a minimal level^40^. Overexpression of NKG2A compromises basic innate responses by exhausting both NK cells and CD8+ cells, whereas several other studies on cancers revealed that blocking NKG2A on those cells restores CD8+ and NK cells functions^41^ and significantly inhibits tumor growth^42^. Studies have shown that pulmonary migration of NK cells and macrophages exert a special role in eliminating SARS-CoV^43^. This innate response promotes a stable immunity against the SARS-CoV regardless of assistance from antibodies or CD8+ cells by producing cytokines and chemokines^31^. A similar approach is in clinical trials in China to unravel whether the same outcome is reproducible in the case of COVID-19 (NCT04280224)^29^. Many companies attempted to treat COVID-19 via repurposed NK-based products. The Kleo Pharmaceuticals from USA, in association with a South Korean Green Cross LabCell company are working together to develop such products. The placental haematopoetic stem cell-derived NK cells, CYNK-001 is developed by a company named Celularity from USA (NCT04365101)^39^.

### 5.2 Chemically Derived Drugs

Chemically derived drugs are comparatively more diverse, as can be determined from this evaluation (**Figure 3**B). The most talked about among them is hydroxychloroquine, an antimalarial drug reported to have more credibility in eliminating the symptoms than any other, although no peer review article has ever confirmed it^44^ as of the time we are writing this review. Even a combination of hydroxychloroquine and some other drugs like azithromycin is being tested on subjects to find out whether the supposed inclination towards this particular drug is to be attended to or not. About 542 chemical drugs are under incessant clinical trials around the world and about 150 of them pertaining to hydrochloroquine, chloroquine and their combinations are under rapid clinical trials, making this a statistically significant investment behind one particular type of drug (**Table 3**). Lopinavir and ritonavir, drug combination used against HIV is also listed as one of the most researched ones. Several antiviral drugs are presented too, with remdesivir, favipiravir being the notable ones. Some of the other chemical drugs which got the most focus are azithromycin, losartan, colchicine, enoxaparin, ivermectin and nitric oxide. Traditional Chinese medicine therapy has also been an initial approach to fight back in China during the onset of the outbreak and is still being researched to this day^45^.

**Table 3:** Clinical trials using chemically derived drugs

Chemically Derived Drugs	Clinical Trials
Hydroxychloroquine	155
Azithromycin	48
Ivermectin	25
Chloroquine	24
Lopinavir/Ritonavir	22
Favipiravir	22
Enoxaparin	19
Nitazoxanide	14
Nitric Oxide	13
Colchicine	12
Ruxolitinib	10
Others	411

#### 5.2.1 Remesivir

Remdesivir has been one of the most controversial entries for clinical drug trials against COVID-19. The drug has shown enough credibility and all-encompassing properties against RNA viruses particularly^46^. It acts as an adenosine analogue which terminates viral replication prematurely^30^. Initially developed to fight against Ebola virus by Gilead Sciences in USA, remdesivir was not proved to be successful but was considered safe for humans that consequently made it a potential candidate for repurposed drug research against COVID-19^47^. Consistent with antiviral activities against SARS-CoV and MERS-CoV^48,49^, the drug was also being considered under clinical trial against SARS-CoV-2^50^. It was first prescribed for the very first reported case in USA on his 7^th^ day in the hospital and he showed a significant improvement the following day with no side-effect^51^. Remdesivir is being experimented on multiple clinical trials across the world in terms of both single trials (NCT04431453, NCT04280705 etc) and in different combinations (NCT04409262, NCT04315948 etc). A randomized, double-blind, placebo-controlled, multicenter clinical trial at ten hospitals in Hubei, China revealed that remdesivir use was not associated with a difference in time to clinical improvement in patients compared to subjects receiving placebo. The US National Institutes of Health (NIH) conducted a double-blind, randomized, placebo-controlled trial of intravenous remdesivir in adults hospitalized with COVID-19 with evidence of lower respiratory tract involvement. Study revealed that those who received remdesivir had a median recovery time of 11 days as compared with 15 days in those who received placebo. The Food and Drug Administration (FDA) had made remdesivir available under an emergency-use authorization for the treatment of adults and children with severe COVID-19 disease in early May. In late May, FDA officially issued a conditional approval for remdesivir in Taiwan and eventually other countries like EU and Canada started to adopt the legal approval for a regular use against the disease^52^.

#### 5.2.2 Favipiravir

The drug favipiravir branded as Avigan was developed by Toyama Chemical (division of Fujifilm, Japan)^53^. Favipiravir follows the mechanism of competitive inhibition to alter the activity of RNA-dependent RNA-polymerase, by resembling the structural makeup of endogenous guanine^54^. The drug is already approved for influenza and has also been designated by National Medical Products Administration in China as the first anti-COVID-19 drug of the country^31^. Repurposing favipiravir for COVID-19 is much of a challenge because of lesser preclinical trials on the drug compared to remdesivir, although both of them are similar in terms of their actions^31^. Number of favipiravir clinical trials (NCT04445467, NCT04434248, NCT04411433 etc) is marching on with increasing expectations. Stanford Medicine researchers launched a clinical trial to test whether an oral drug can reduce symptoms and viral shedding in people with COVID-19 beginning in July 6^55^. Simultaneously, Glenmark is conducting phase III clinical trials of favipiravir as a COVID-19 monotherapy option with 150 patients, enrolled from 9 leading government and private hospitals across India.

#### 5.2.3 Lopinavir/Ritonavir

A popular drug combination of lopinavir and ritonavir against human immunodeficiency virus (HIV), which is basically a facilitator of protease inhibition in the virus itself, already made its mark as a successful therapeutic agent^31^. Ritonavir combined with lopinavir leads to increased plasma half-life. Such combination was reported to lead to decreased adverse results in the 2004 SARS outbreak^30^. Studies showed that they can work as inhibitors to the cysteine protease that coronaviruses encode^56-58^. Experimental and clinical results proved combination to be effective against both SARS and MERS-CoV^50,51,54,59^. Hence, the combination of lopinavir and ritonavir was repurposed against the SARS-CoV-2 (NCT04252885) as well. However, only a small improvement was noticed in mild cases^49^ whereas nothing remarkable could be extracted in the severe ones^60^. Earlier study in China found faster clearance of SARS-CoV-2 by PCR and faster improvement of chest computed tomography. A randomized, open-label clinical trial comparing LPV/r versus placebo in patients with severe COVID-19 found no statistically significant difference in time to clinical improvement or 28-day mortality. In July, WHO announced to discontinue lopinavir/ritonavir treatment for COVID-19, since clinical trial results showed that hydroxychloroquine and lopinavir/ritonavir produce little or no reduction in the mortality of hospitalized COVID-19 patients when compared to standard of care.

#### 5.2.4 Hydroxychloroquine

Hydroxychloroquine, most famously known as an anti-malarial drug and an anti-immune agent operates by suppressing acidification of cellular endosome during the events of replication and infection^61^. As for the case of SARS-CoV-2, it has shown its ability to inhibit the replication of this virus as well by interrupting the glycosylation of its receptor protein-ACE2^62^. This claim has been supported by several in-vitro clinical trials of the drug that saw a significant reduction in the copy numbers of SARS-CoV-2^63^. Clinical trials in China concluded that hydroxychloroquine is only effective against COVID-19 associated pneumonia, not against the virus specifically^31^. A non-randomized clinical trial in France gave out a somewhat optimistic result when hydroxychloroquine was combined with azithromycin (NCT04339816, NCT04335552)^64^. However, the WHO decided on July 15, 2020 to halt clinical trials of hydroxychloroquine as a potential treatment for hospitalized COVID-19 patients^65^. When compared with the standard of care in the treatment of hospitalized COVID-19 patients, hydroxy-chloroquine did not result in the reduction of the mortality of the patients.

#### 5.2.5 Arbidol (Umifenovir)

Arbidol acts upon influenza viruses and arboviruses as an entry inhibitor^66^. It interrupts the endocytosis of influenza virus by preventing the fusion of viral body with endosome through targeting hemagglutinin of viral surface^31^. Arbidol is already approved in Russia and China for COVID-19 drug trials now as a single agent^66^. But in a comparative study, favipiravir turned out to be more effective than arbidol^67^. An in vitro study in China shows that arbidol inhibits SARS-CoV-2 infection whenever applied at a minimal concentration. Nowadays, a dose of (200 mg) arbidol is given three times per day not for more than 10 days to treat COVID-19. A randomized study was also conducted to determine the efficacy of arbidol in combination with recombinant human interferon-α2β to treat mild to moderate pneumonia due to COVID-19^68^. China awaits two phase IV drug trials of Arbidol (NCT04260594, NCT04286503), to provide a conclusive result by February 2021.

#### 5.2.6 Angiotensin Receptor Inhibitor

For an angiotensin receptor inhibitor, recombinant human angiotensin converting enzyme 2 (rhACE2) is now being recommended because of its ability to block S protein from binding to the host receptor ACE2^31^. In fact, rhACE2 is found to be able to obstruct SARS-CoV-2 replication by a factor of about 5,000 times maximum according to contemporary studies on embryonic cell-mediated organoids^69^. Applying rhACE2 has been reported to abate serum level of angiotensin 2 that diverts the substrate away from the receptor ACE; eventually intervening with activation of ACE2 as well to prevent ARDS (Acute Respiratory Distress Syndrome)^70^. In China, a study has been carried out to evaluate the biological and physiological role of rhACE2 NCT04287686) against COVID-19 and so it made to the list of potential drugs against SARS CoV-2 in terms of measurement of plasma level of angiotensin 1-7^31^. A couple of other studies in Austria and Australia (NCT04353596, NCT04394117) are dealing with the final phase of angiotensin inhibitory drugs due to be published in two years.

#### 5.2.7 Thalidomide

Thalidomide was used as a treatment plan against some of the severe inflammatory diseases such as Behcet’s disease and Crohn’s disease and it acts by decreasing synthesis of TNF-α^71^. Studies also reported the successful use of thalidomide against H1N1-infected mice, as it facilitates production of pro-inflammatory cytokines and reduction of infiltration of inflammatory cells^72^. Recent emergence of thalidomide as an anti-inflammatory, anti-fibrotic and anti-angiogenic agent has put it in the discussion of being a repurposing drug against certain SARS-CoV-2 strains (NCT04273321, NCT04273581) predicting its immunomodulatory ability of reducing lung defect^31^.

#### 5.2.8 Methylprednisolone

Pathogenesis of SARS-CoV-2 is supported not only by the direct damage it causes to the host, but also by the excessive immune response that the host cell offers^31^. Methylprednisolone is one of the potential drugs like thalidomide that can supposedly inactivate such unexpected immune reaction. Nowadays along with antibiotics, oseltamivir, and oxygen therapy methylprednisolone is being used to treat COVID-19 patients^33^. Several clinical trials are ongoing (NCT04263402, NCT04355247, NCT04343729) to determine whether or not it’s safe and useable^31^.

#### 5.2.9 Chloroquine

Chloroquine is an anti-malarial drug^73^. While it has been used as an effective anti-viral drug, as of now it is being considered to be repurposing against COVID-19^74^. Chloroquine inhibits pH-dependent steps of the replication of many viruses that has already been quite extensively tested both *in vitro* and *in vivo* on different virus strains and recently, on SARS-CoV-2. Though treatment with chloroquine showed promising results, it also strongly differed in application between live animals and cell lines. The major finding was that even if chloroquine shows promising results on virus^75^ and cells, the in vivo application is not so promising^75^. China, South Korea and Italy allowed for experimental trials of chloroquine against SARS-CoV-2^76,77^. Having a lower therapeutic index, chloroquine may often turn out to be toxic for the user^78,79^. FDA didn’t approve hydroxychloroquine and chloroquine under emergency use authorization (EUA)^80^. In short, no conclusive clinical trial has proved efficacy of the drug till now although research is still underway in Mexico (NCT04323527, NCT04342650), Egypt (NCT04353336), USA (NCT04349371), UK (NCT04303507) and many other countries.

#### 5.2.10 Losartan

Losartan is a widely used drug for treating hypertension and left ventricular hypertrophy (heart muscle enlargement) and kidney dysfunction in type-2 diabetes^81^. It blocks the receptor of angiotensin-2 from entering the cells and raising blood pressure. Losartan is being perceived as an antagonist, since it interferes with the substrate-ACE2 receptor interaction of SARS-CoV-2^82^. However, according to a recent study, such medication for hypertension might end up inducing the host to produce more ACE2 instead and increasing susceptibility of the virus in the process^83,84^. Various studies are taking place across different phases of development on losartan including a phase IV study by Sharp Healthcare (NCT04340557) and a couple of phase II studies by University of Minnesota (NCT04312009, NCT04311177).

#### 5.2.11 Azithromycin

Azithromycin could reduce the severe lung inflammation caused by SARS-CoV-2 infection, through halting the production of cytokines, along with building immunity against other viruses. Azithromycin along with hydroxychloroquine was able to inhibit SARS-CoV2 replication in a clinical trial in France, however, the result was not reproducible when the antibiotic was administered alone^85^. Few phase III studies are also being carried out in France solely on azithromycin (NCT04371107, NCT04365582). Gautret et al. reported a 100% viral clearance in nasopharyngeal swabs in their 6 patients whenever they were treated with both the drugs. The findings reported by Molina et al. stood in contrast with those reported by Gautret. They repeated the experiments and found 8 of 11 patients having remarkable comorbidities^83^.

#### 5.2.12 Ruxolitinib

Ruxolitinib is used to treat intermediate or severe myelofibrosis (bone marrow disorder)^83,86^ and polycythemia vera (PCV) that occurs when there’s a lack of response to hydroxyurea^87^. It is a janus kinase inhibitor (JAK inhibitor) particularly involving JAK1 and JAK2^88^. It was reported to cause faster improvement of the COVID-19 patients by Ruxolitinib administration but a phase II clinical trial failed to account this^89^. Incyte, a biopharmaceutical company, has initiated a phase III clinical trial to assess the safety and efficacy of the drug material (NCT04377620)^90^. Few other phase II and phase II / phase III clinical trials are focused on ruxolitinib in both America and Europe (NCT04348071, NCT04338958, NCT04359290).

#### 5.2.13 Baricitinib

Baricitinib is another anti-inflammatory drug approved to be used against rheumatoid arthritis (RA), as a janus kinase inhibitor (anti-JAK)^91^. The anti-inflammatory properties of baricitinib inspired some groups to observe its effect in inflammation^92^. Oral administration of baricitinib could potentially reduce inflammation in COVID-19 associated ARDS^93^. National Institute of Allergy and Infectious Diseases (NIAID) launched a study on combination of remdesivir and baricitinib for a better anti-inflammatory outcome than remdesivir alone (NCT04401579)^93^. As for single studies, baricitinib is currently being researched in various clinical trial phases (NCT04340232, NCT04399798 etc).

#### 5.2.14 Nitric Oxide

Nitric oxide (inhaled nitric oxide (iNO)) produced by Mallinckrodt Pharmaceuticals Inc is known to reduce pulmonary hypertension and hypoxia by lessening ventilation support^94^. A phase II study is being conducted on iNO in patients afflicted with ARDS resulted from SARS-CoV-2 infection^95^. A phase III study has also been approved by FDA for ensuring safety and efficacy of the drug in patients with COVID-19 needing supplemental oxygen before advancing to forced ventilation^95^. On March 20, 2020, FDA granted emergency expanded access allowing its iNO delivery system (INOpulse) to be immediately used for the treatment of COVID-19 (NCT04421508). However, more study results are needed to be evaluated (NCT04212243, NCT04398290) to determine the efficacy of iNO in treating COVID-19 patients.

#### 5.2.15 Vitamin C

Vitamin C also known as ascorbic acid helps boost the immune system through its antioxidant properties and collagen synthesis. It is an effective and simple molecule without any side effects or cross-reaction with available COVID-19 drugs. Some hospitals have been reported to have prescribed high-dose of intravenous vitamin C for the treatment of 50 moderate to severe COVID-19 patients in China. The dose varied between 2 and 10 g per day over a period of 8 to 10 hours of IV (intravenous) infusion. The oxygenation index was improved and all the patients recovered^84^. A study confirmed the ability of vitamin C to lessen death rate by 20% in patients with lung damage^96^. Another study in China showed zero death out of 50 intermediate patients treated with high doses of vitamin C. Progenbiome of USA has initiated a couple of phase II clinical trials on Vitamin-C (NCT04334512, NCT04335084) alongside other drugs. A phase III research on only Vitamin-C has also been brought under consideration in Sherbrooke University (NCT04401150, NCT03680274). However, the overall effect and outcome of vitamin C in treating COVID-19 alone or in combination are disputable.

#### 5.2.16 Dexamethasone

Dexamethasone is a corticosteroid used for different inflammation conditions and other diseases like arthritis, lupus, breathing disorders etc. A study in March 2020 concluded that an early dose of dexamethasone would reduce inflammation in COVID-19 associated ARDS and thus control mortality rate to a large extent suppressing ventilation tendency^97^. Recently, dexamethasone proved to be the first life-saving drug among seriously ill COVID-19 patients. The study comprised of 2,100 candidates receiving dexamethasone compared with 4,300 people given standard care. Dexamethasone reduced death by one third for people on ventilation and reduced risk by one fifth for the patients needing oxygen. But the results have not yet been peer reviewed. Dexamethasone phase III and phase IV clinical trials to conclude in near future will be able to help create an extensive platform for further utilization of the drug (NCT04325061, NCT04395105, NCT04327401 etc).

#### 5.2.17 Oseltamivir

Oseltamivir is an already approved drug against influenza A and B^20^ that targets the neuraminidase on the surface of influenza virus to inhibit the virus from entering human cells^98,99^. An initial clinical trial in Wuhan didn't show a success when using the drug for COVID-19 patients. Still, oseltamivir is being studied in a combination with chloroquine and favipiravir around the world. A phase III clinical trial on oseltamivir by Federal Task Force on Science and Technology in Pakistan (NCT04338698) is expected to provide a definitive take on the future of this particular drug.

#### 5.2.18 Colchicine

Colchicine is an anti-inflammatory drug used for gout management along with treating other complications^20^. It blocks neutrophils from transferring to inflammation sites and inhibits forming inflammasome complex blocking its activation^100^. Colchicine has been reported to reduce inflammation in the cardiac myocytes of patients with both COVID-19 and myopathies^101^. Several other studies are being conducted on colchicines, NCT04355143, NCT04322682) for cytokine storm^20^.

#### 5.2.19 Nitazoxanide

Nitazoxanide is an anti-viral drug inhibiting the expression of viral N protein, shown to have inhibitory effects against MERS-CoV in LLC-MK2 cells, along with several other coronaviruses, mouse hepatitis virus strain A59 (MHV-A59), human enteric corona virus 4408 (HECoV-4408) and bovine coronavirus strain L9 (BCoV-L9)^102^. Through an unreliable source, it is also reported that the drug suppresses proinflammatory cytokines in peripheral blood mononuclear cells (PBMC) and IL-6 in vivo^103^. Nitazoxanide 500 mg is in phase IV clinical trials due to be concluded by the end of the current year (NCT04341493, NCT04406246).

#### 5.2.20 Selinexor**

Selinexor is an FDA approved drug in high doses, used for cancer conditions, such as relapsed multiple myeloma. Manufactured by Karyopharm Therapeutics Inc, selinexor is an oral selective inhibitor of nuclear export (SINE) in cells^104^. It has been seen that blocking of nuclear export automatically disrupts the replication of SARS-CoV-2. With that in mind, selinexor is now being explored through phase I and phase II clinical trials, by leading health organizations like Norton Healthcare and Lehigh Valley Health Network. Currently, Karyopharm therapeutics is conducting a phase II clinical trial on the drug (NCT04355676, NCT04349098).

#### 5.2.21 Methothrexate**

Methothrexate (MTX) is a drug dedicated to treat different cancer cases, such as breast, skin and lung cancer and to treat psoriasis and rheumatoid arthritis in lower doses. While it is still not known whether SARS-CoV-2 is susceptible to the drug or not, for those COVID-19 patients with rheumatoid arthritis as an additional condition it is recommended to stop their medication on MTX for a while until the complexity is resolved. A Brazilian research team collaborating with an Indian-based contract research organization has initiated a study on methotrexate nano-particles (NCT04352465) to treat COVID-19 by evaluating inflammatory responses in patients^105^.

#### 5.2.22 Tranexemic Acid**

Tranexemic acid or TXA is a derivative of amino acid lysine helping blood coagulate. It carries antifibrinolytic property to reverse excessive blood loss from traumas and surgeries. One of the lesser known symptoms of COVID-19 is coagulopathy. University of Alabama researchers are conducting two phase II clinical trials on TXA (NCT04338074, NCT04338126), to figure out its ability in balancing the coagulation factors in COVID-19 patients^106^.

#### 5.2.23 Traditional Chinese Medicine**

Traditional Chinese Medicine (TCM) which uses phytotherapeutic formulations like teas, pills, powders or tinctures, and cultural components can be traced back 5000 years ago in Chinese medicine. These were already used for SARS-CoV infection as coadjuvant therapy with the enhancement of patient’s symptoms, increased oxyhemoglobin arterial saturation in 2002^33^. TCM synthesized by local Chinese manufacturers claimed to have effect in combating infectious diseases such as COVID-19.

## 6. Vaccines

With the rising cases of SARS-CoV-2 infection, it is apparent that COVID-19 is going to be prevailing for a long time and the battle against any virus can be ultimately won through an effective and safe vaccine^107^. The first vaccine (Pfizer-BioNTech mRNA-based vaccine) just received emergency approval in December 2020 for use in USA and UK. Additional vaccines (such as the mRNA-based vaccine from Moderna) will follow. These are excellent news. However, it will take months to have the vaccines distributed worldwide, their long-term effects are hard to predict, and the possibility of future resistant mutations is a concern.

**Figure 4:**
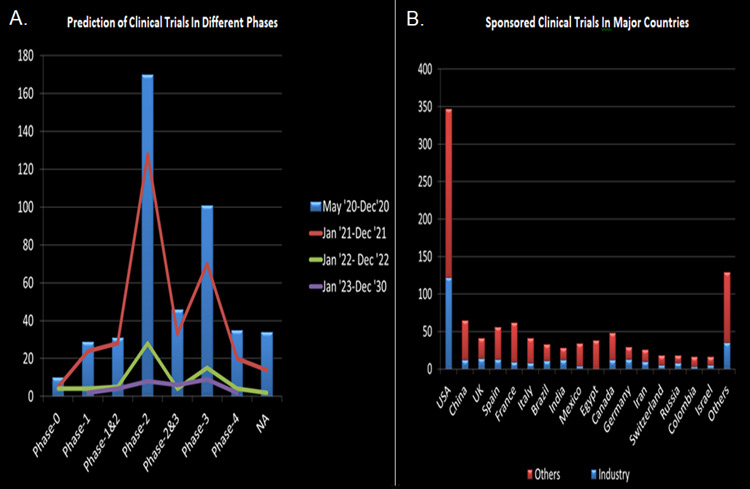
A graphical representation of (A) leading countries and sponsors associated with the clinical trials of the COVID-19 drug candidates and (B) phases and predicted timeline of the clinical trials of the COVID-19 drug candidates Many countries with at least 10 ongoing clinical trials through different sponsorships are shown by their names. Sponsors are categorized into two distinct groups: industry and others that include universities, organizations, government and government agencies. Different phases of drug trials with respect to specific timelines of potential approval are presented. Some of the drugs are in simultaneous clinical trials of two different phases, whereas the specific phases are not mentioned clearly for some others, hence kept in NA (Not applicable). The graphs have been generated using the Microsoft Excel application.

Vaccines can be a reliable therapeutic approach^108^ by promoting a long term strategy against the virus^39^. Experts have warned of any shortcut in vaccine development due to public pressure which may elicit antibody-dependent enhancement, resulting in long term consequences^109^. In spite of these, we really cannot rule out the fact that production of vaccine is the most effective and viable alternative to combat an emerging pandemic, such as COVID-19. Many vaccine clinical trials are currently going across the globe, including a BCG vaccine (NCT04348370, NCT04417335), heat killed Mycobacterium vaccine (NCT04347174, NCT04358809) and MMR vaccine (NCT04357028). ChAdOx1 ncoV-19 (NCT04444674), recombinant nCoV-19 (NCT04313127) and inactivated nCoV, which are in different clinical phases now to develop host immunity (**Figure 4**A). We are discussing several COVID-19 vaccines in clinical trials below.

mRNA-1273 (ClinicalTrials.gov number, NCT04283461), developed by Moderna, an American biotech company in association with National Institute of Allergy and infectious disease (NIAID), has caught the scene for a while now. It is a synthetic mRNA which is surrounded by minute particles composed of lipids. Similar to the Pfizer-BioNTech vaccine (which is also an mRNA-based vaccine), it is supposed to induce antiviral response in the injected cells against spike proteins of SARS CoV-2 (NCT04283461)^39,110^. No virus was required to formulate the vaccine and platform technology made its production easier and safer for testing (NCT04283461)^39,109^. Elevated titer of neutralizing antibody was observed in convalescent serum in all the initial 45 candidates whenever 2 dosages were applied of that particular vaccine^111^. Moderna is very much hopeful that mRNA-1273 will be commercially available on the market soon. The study group published a preliminary report on July 14, 2020 of a phase I, dose-escalation, open-label clinical trial including 45 healthy adults, 18 to 55 years of age, who received two vaccinations, 28 days apart, with mRNA-1273 in a dose of 25 μg, 100 μg, or 250 μg. The mRNA-1273 vaccine induced anti-SARS-CoV-2 immune responses in all participants, and no clinical trial-limiting safety concerns were identified. These findings shed light of hope to the already COVID-19 stricken world.

INO-4800, a DNA vaccine developed by INOVIO Pharmaceuticals (NCT04336410, NCT04447781) is another genetic vaccine that utilizes the process of immune response against specific viruses. Such genetic vaccines do not involve risk of improper protein folding like the protein-based vaccines^34,112^. The INO-4700 was administered successfully against MERS-S within a DNA vector and INO-4800 is now in clinical trials against SARS-CoV-2-S^31^. INO-4800 is transdermally applied to healthy candidates through the help of an electroporation device called Cellectra, to determine its immunological features^111^. Ease in purification and lower expense in production make it potentially preferable over other vaccine types^39^. In preclinical animal challenge study, INO-4800 provided full protection against SARS-CoV-2 replication in the lungs in mice. 94% of participants demonstrated overall immune responses at week 6 after two doses of INO-4800 in phase I clinical trial with 40 healthy volunteers. The vaccine elicited no serious adverse effects through week 8 with some grade I severity.

ChAd vectors have already been established as safe and 100% efficient with single vaccination^113^ against viruses like Ebola virus^114^, Influenza A^115^ and MERS^116^. ChAdOx1 vaccine was designed by the University of Oxford in collaboration with AstraZeneca, based upon adenovirus vector against the spike protein S of SARS-CoV-2 (NCT04324606)^39^. ChAdOx1 vaccination prevented lung damage, indicating promising outcomes in the near future. The vaccine began phase I clinical trial in April. The University later went on to host two more clinical trials on ChAdOx, including one in collaboration with South Africa (NCT04444674, NCT04400838). With all these being said, if successful, ChAdOx is showing immense probability of being one of the most sustainable vaccines against COVID-19, although if the induced immunogenicity in host is propelled towards the vector genome rather than the transgenes, it might cause an unexpected failure in the vaccination process. Over 5,000 healthy volunteers began participating in Brazilian clinical trial of this vaccine. Oxford researchers have begun recruiting for the phase II/phase III clinical trial and planning to apply ChAdOx1 on children.

Nanoparticles-based vaccines are produced through encapsulation by adjoining with antigenic epitopes mimicking viral attacks^39^. General nanoparticles-based vaccination includes oral and intranasal uptake, which can induce immunity on the mucosal surface, in addition to the systemic immune responses^117^. This indicates how these vaccines would be effective against respiratory viral infections, including SARS-CoV-2^39^. An investigation was conducted to determine potency between nanoparticle (polyethylenimine) that elicited spike (S) protein of SARS-CoV and naked plasmid vector. Nanoparticle produced elevated level of spike (S) protein specific antibody over naked plasmid vector^118^. For that purpose, Novavax Inc. has taken the initiative of producing a vaccine based on nanoparticles using spike proteins of SARS-CoV-2 (NCT04368988)^39^. Another study is under clinical trial as of now evaluating mRNA-lipid nanoparticles-based vaccine previously successful against SARS and MERS-CoV (NCT04283461). Many additional vaccines are now in clinical trials and expected to be approved for use in the near future.

## 7. Countries and Sponsors Involved in Drug Trials

The United States is dominating the ranking for the number of clinical trials for the highest number of drugs (347). As far as drug research is concerned, in China, they have 65 drug trials ongoing as of now (**Figure 4**B). France and Spain come next with 62 and 56 ongoing clinical drug trials respectively. Among other most affected nations, Italy has 41 drug trials ongoing, whereas Canada holds about 48 clinical trials attempting to battle COVID-19 (**Table 4**). It can be derived that North American countries have an upper hand in drug research, whilst other than China, the Asian countries are lagging quite behind. European countries are also providing a decent and above average service in developing drugs. Most of the drug trials are being sponsored by various universities and organizations, while multiple pharmaceutical giants are also investing in a significant number of clinical trials across the globe. There are few other government sponsored organizations, such as NIH and FDA investing in a number of clinical trials.

**Table 4:** List of countries affiliated with drug R&D with corresponding number of clinical trials through industrial and other (universities and other academic institutions, government funding and other government agencies, e.g. NIH) interventions

Country	Industry
USA	122
China	12
UK	14
Spain	13
France	9
Italy	8
Brazil	11
India	12
Mexico	4
Egypt	0
Canada	12
Germany	13
Iran	10
Switzerland	5
Russia	8
Colombia	3
Israel	5
Others	35

## 8. Timeline and Clinical Trial Phases

Each of these tested drugs is constantly going through clinical trials and errors to be finally selected for approval. Most of the drug trials are in developmental phase II and III (335 and 195 respectively). 61 drug trials are in the final phase of drug development, while 50 others have no designation at all as in what phase they are in (**Figure 4**B).

There is no telling as to when a suitable drug may be advertised to cure COVID-19. But as it is estimated, among the 876 clinical drug trials, over half of them may be well predicted by the end of 2020 (**Table 5**). However, gaining a positive result out of them still asks for absolute confirmation of the feasibility of the drugs from clinical trials on population. That may lead to some of the drugs taking up to 2030 to be finally prescribed as safe. Since the majority of the drugs are currently nearing to the final phase of development, it may be expected that within late 2021, sustainable drug products will be well constructed and readily available or will lay out some standard and similar protocols for an effective drug at the least.

**Table 5:** Number of ongoing clinical trial phases of drugs in terms of a specific timeline

Phases	May '20 – Dec '20	Jan '21-Dec '21	Jan '22- Dec '22
Phase-0	10	5	4
Phase-1	29	24	4
Phase-1&2	31	28	5
Phase-2	170	128	28
Phase-2&3	46	33	4
Phase-3	101	70	15
Phase-4	35	20	4
Not applicable	34	14	2

## 9. Conclusion

Worldwide researchers and physicians are doing their best to extract more information about the virus, so that they can figure out effective ways to completely eliminate the lethal pandemic, which has become a threat for human health worldwide. In such emergence of the pandemic, drug repurposing is being considered to be a quicker way to show rapid and effective response. This study tried to systematically address the current scenarios of the clinical trials of the repurposed drugs. Numerous repurposing drug candidates are in clinical trial in many countries simultaneously. Several biological treatments, including convalescent plasma, recombinant interferon, mesenchymal stem cell, antibodies, and the use of the NK cells are undergoing clinical trials and has already exhibited promising results. Among chemically derived drugs, remdesivir was the first treatment for COVID-19 to receive FDA approval. Dexamethasone proved to be the first life-saving drug among seriously ill COVID-19 patients. For hydroxychloroquine, despite showing some promising results at first, clinical trials were later halted at WHO’s recommendation. Although further investigations are needed to be carried out, there is a high probability that within a short period of time, some of these various treatment plans in clinical trials will be approved in terms of safety and efficacy and will be available in the global market at lower cost. Such efforts may facilitate the discovery of new classes of medicines as well. While attempts to synthesize a novel treatment plan continue from the scientific community, the repurposing treatments in clinical trials are bound to provide a reliable ground for withholding the disease until that novelty is achieved. In addition, some of the novel vaccine candidates in clinical trials are already listed here and many more are in the pipeline to undergo clinical trials soon. Such a detailed view of drug and vaccine candidates is expected to assist in and add a new dimension to the ongoing process of developing therapeutic and prevention options against COVID-19, on a huge global platform.
